# A systematic review comparing caregiver burden and psychological functioning in caregivers of individuals with schizophrenia spectrum disorders and bipolar disorders

**DOI:** 10.1186/s12888-022-04069-w

**Published:** 2022-06-23

**Authors:** George J. Karambelas, Kate Filia, Linda K. Byrne, Kelly A. Allott, Anuradhi Jayasinghe, Sue M. Cotton

**Affiliations:** 1 Orygen, 35 Poplar Road, Melbourne, Victoria 3052 Australia; 2 Centre for Youth Mental Health, University of Melbourne, Melbourne, Australia; 3 School of Psychology, Deakin University, Melbourne, Victoria Australia

**Keywords:** Caregiver, Caregiver burden, Bipolar disorder, Schizophrenia Spectrum disorders, Mental health

## Abstract

**Background:**

Informal primary caregivers provide crucial supports to loved ones experiencing serious mental illnesses with profound outcomes for the caregivers themselves. A comprehensive understanding of how different serious mental illnesses change the caregiving experience may provide important insight into the ways in which caregivers can be better supported in their role. The aim of this review was to synthesize the comparative literature examining caregiver burden and psychological functioning (anxiety, depression, distress, and psychological wellbeing) between caregivers of people with schizophrenia spectrum disorders and bipolar disorder.

**Methods:**

Studies were included if they compared caregivers across both diagnostic groups and used measures assessing either caregiver burden or psychological functioning of caregivers. Databases searched up until 11th of January 2022 included: Medline COMPLETE, Embase, PsycINFO and CINAHL. Reference list scans and grey literature searches across government, organisational and dissertation databases were also conducted.

**Results:**

Twenty-eight studies comprising 6166 caregivers were included. Fourteen studies suggested that caregiving burden was comparable across both groups. The effects of caring on caregiver mental health and stress were comparable across both groups. However, methodological limitations were noted, including a reliance on cross-sectional studies, multiple and sometimes competing definitions of caregiving burden, variable sample sizes, and variation in measures used.

**Conclusion and implications:**

The experience of providing care is multidimensional and complex. Symptoms and functional difficulties experienced by people being cared for may affect caregivers more so than diagnosis. Caregivers play a vital role in helping people with serious mental illness. Supporting caregivers by reducing their burden and improving their psychological functioning may help them to continue to provide support, and cope with, the challenges of providing care.

## Introduction

Serious mental illnesses (SMI), such as schizophrenia spectrum disorders and bipolar disorder, incur significant functional impairments [1, 2], increase the likelihood of disability [3, 4], and shorten life expectancies [5]. The severity and chronicity of SMI results in associated care costs of $56.7 billion to the Australian healthcare system [6]. Personal costs associated with supporting individuals with SMI are often experienced by informal primary caregivers (hereafter referred to as ‘caregivers’), who are family members, spouses, friends, or close others who provide primary support [7]. Caregivers assist with emotional support, daily living, finances, behavioural management, liaising with professionals, and functional recovery [8]. The global move away from institutionalization and greater reliance on caregiver supports has resulted in better outcomes for individuals with SMI in terms of symptomatology and quality of life [9]. However, the increasing reliance on caregivers, whose role has an approximate annual economic value of $13.2 billion in the Australian mental health system [10], has profound impacts on caregivers themselves. These impacts have often been considered with relation to caregivers of those with medical conditions such as cancer or dementia [11–13]. However, increasing focus has been placed upon caregivers of those with SMI, and several outcomes of caring have been investigated, with caregiving duties negatively associated with caregiver physical health [14], financial burden [15], rates of employment [9] and quality of life [16, 17].

Caregiver burden, or the difficulties experienced in providing care, is one outcome frequently examined [18]. There exist several conceptualisations of caregiver burden, however the most common terms are objective and subjective burden [19]. Objective burden represents observable and verifiable disruptions from providing care, whereas subjective burden represents personal feelings of burden [20]. As caregiver burden encompasses several difficulties of providing care, the psychological functioning of caregivers has also been considered.

Psychological functioning, or the ability of individuals to achieve their aspirations in their external environment [21], includes mental health difficulties caregivers may experience, like anxiety, depression and caregiver distress. Anxiety and depression are typically assessed by symptoms such as worried thoughts, feelings of worthlessness, diminished interest in daily activities, and disruptions to sleep and energy [22]. Caregiver distress represents emotional suffering, characterised as loss, hopelessness or restlessness, in response to specific stressors that affect caregivers [23, 24]. Psychological wellbeing, a component of psychological functioning, is a concept that has seldom been considered in caregivers. It can be divided into hedonic and eudaimonic wellbeing. Hedonic wellbeing represents feelings of short-term happiness and positive emotions [25], whereas eudaimonic wellbeing refers to one’s life purpose and self-acceptance [26]. Given the vital role of caregivers, considering their psychological functioning from this multidimensional perspective can inform the available supports caregivers require while caring for loved ones with SMI.

Most research of SMI has involved examinations of caregiver burden and psychological functioning for caregivers of those with schizophrenia spectrum disorders, and more specifically, chronic schizophrenia [16]. This is understandable, given schizophrenia spectrum disorders are defined by significantly impactful positive symptoms, such as hallucinations and delusions, alongside significantly impactful and often persistent negative symptoms, including social withdrawal [22]. Individuals with these disorders are at greater risk of poorer outcomes, including early mortality in comparison to the general population [27]. Caregivers of those with schizophrenia have reported high subjective burden [28], and the severity of positive psychotic symptoms has been associated with significant objective burden relating to financial demands, increase in family conflicts and mental health difficulties [29], and decreases in life satisfaction [8]. However, focusing on caregivers of those with chronic schizophrenia limits the generalisability of findings for caregivers of those with other diagnoses of SMI and at earlier stages of caregiving. SMI diagnoses may be associated with differential experiences of caregiving due to differences in symptoms and associated impacts, whereas the stage of diagnosis may reflect differences in the caregiver’s adjustment and appraisal of their loved one’s illness. By understanding whether differential outcomes exist, the treatments and supports for caregivers can be specified to reflect the circumstances of their caring role. In doing so, this may benefit caregivers more so than a generalized approach to caregiver supports. Additionally, the focus on negative caregiver outcomes means there has been lesser focus on the psychological wellbeing of caregivers. This field of literature is growing [30, 31]. However, further research is necessary in considering the potential for positive growth and improved wellbeing in association with providing care. These factors may help reduce the level of burden and mental health difficulties reported by caregivers.

A growing number of studies have compared how different SMIs affect caregiving, particularly for caregivers of those with bipolar disorder [32–34]. Bipolar disorder contrasts from schizophrenia spectrum disorders as it is characterized by symptoms that reflect significant shifts an individual’s energy, mood and activity [22]. All-cause mortality rates, representing death by any cause, in populations diagnosed with bipolar disorder is double the rate found within the general population [35]. While bipolar disorder presents with differing symptoms to schizophrenia, the rates of mortality between both disorders have been found to be similar [36]. Caregivers of those with bipolar disorder report severe burden and heightened anxiety, depression and distress during their caregiving experience [37, 38]. The literature for caregivers of those with bipolar disorder presents with similar limitations to that of the literature for caregivers of those with schizophrenia – primarily, the focus on chronic presentations and relative paucity of psychological wellbeing literature. Despite these limitations, the burden and mental health difficulties that caregivers for those with bipolar disorder experience has often been likened to that of caregivers for those with schizophrenia [39, 40]. These disorders, however, present with differential illness trajectories and symptom profiles [22]. It is possible that caregivers may encounter unique challenges given these diagnostic differences, and several studies have moved to compare whether caregiving outcomes are influenced by diagnosis.

To date, there has been no comprehensive review of these comparative studies to determine whether a clear indicator of how diagnosis may influence caregiving outcomes exists. Prior systematic reviews have examined the impact of schizophrenia [8, 41] and bipolar disorder [42, 43] on caregiver outcomes separately, and others have examined the multidimensional impact of SMI as a broad categorization on family members [44, 45]. None have directly examined the comparative literature to determine whether specific disorder characteristics differentiate caregiver experiences. This review will contribute to the understanding of how disorder characteristics are associated with caregiving, which can help determine optimal support options in facilitating caregiver wellbeing and allowing caregivers to continue providing essential supports.

The aim of this systematic review was to address this gap in the literature and determine whether different diagnostic groups influence the outcomes of caregiving on caregivers. Specifically, we wanted to determine whether caregivers of individuals with schizophrenia spectrum disorders differ from caregivers of individuals with bipolar disorder with respect to:

(i) *caregiver burden*
(ii) *mental health outcomes, encompassing anxiety, depression, and caregiver distress*
(iii) *psychological wellbeing*

## Method

### Protocol

The review protocol was registered and is accessible on The International Prospective Register of Systematic Reviews (PROSPERO; Protocol ID CRD42019120815).

### Inclusion and exclusion criteria

Studies were included in the review if they met the following criteria:

- Published from January 1st, 1900 to January 11th, 2022;
- Presented quantitative data comparing caregiving outcomes between caregivers of individuals with schizophrenia spectrum disorders and bipolar disorder;
- Included samples of caregivers as per criteria specified by Pollak and Perlick [7]. That is, family members satisfying three of the following criteria, or non-family members satisfying two of the following criteria: i) is a spouse (or equivalent), parent, sibling, or close other; ii) has the most frequent contact with the individual; iii) helps to support the individual financially; iv) has most frequently been a collateral in the individual’s treatment, and v) is contacted by treatment staff in case of emergency;
- Included a measure of caregiver burden or psychological functioning (mental health outcomes of caring or psychological wellbeing):
  (i) Caregiver burden measures assessed the difficulties caregivers experience caring for family members with mental illness [18]
  (ii) Mental health outcome measures assessed anxiety, depression or caregiver distress experienced in response to stressors [23]
  (iii) Psychological wellbeing measures were those that assessed hedonic and eudaimonic wellbeing [25, 26].

To ensure saturation of literature, numerous study design types were considered, including observational studies, prospective and retrospective cohort studies, case-control, cross-sectional, and descriptive studies. Baseline data from randomised controlled trials (RCT) comparing caregiving burden or psychological functioning were also considered.

### Exclusion criteria

Studies were excluded if they only contained qualitative data and/or were not in English.

### Identification and selection of studies

Comprehensive literature searches were conducted across electronic databases including MEDLINE Complete, Embase, PsycINFO and CINAHL. Terms and synonyms related to the caring role (i.e., caregiver, carer, support, caring), caring outcomes (i.e., burden, distress), individuals who may be caregivers (i.e., family, parent, sibling, relatives, spouses, partners, grandparents, children, guardian), bipolar disorder (i.e., bipolar I, bipolar II, mania, bipolar affective disorder, manic episode, manic depression), and schizophrenia spectrum disorders (i.e., schizophrenia, psychosis, psychotic, schizophreniform, schizoaffective, delusional disorder) were used (a full electronic search strategy is available upon request). Medical subject headings and controlled vocabularies were applied across all databases. An initial search was conducted on 1st of July 2019 and a final updated search on January 11th, 2022. Authors of articles were contacted where further information was required. Searches for unpublished or non-commercial documents (grey literature) with quantitative data were conducted across the Australian Bureau of Statistics, Australian Institute of Family Studies, Australia Institute of Health and Wellbeing, Analysis and Policy Observatory, Carers Australia, Deakin Library Catalogue (Dissertations), PsycEXTRA, TROVE and Web of Science (Dissertations). Variations of the primary search strategy were implemented depending on the search functionalities of each database. Consistency across databases was maintained by using the search terms focused on “caregivers”, “bipolar disorder” and “schizophrenia”.

### Study selection

All search results were examined using Covidence Systematic Review Management tool [46]. Two authors (GK & AJ) independently conducted duplicate screening. Title and abstracts of each article were independently screened to determine full-text eligibility. All reasons for excluding articles were noted. Full-text screening of included articles was again independently conducted by GK & AJ. To ensure literature saturation, the reference list of all articles included in the final systematic review were scanned for additional studies.

### Data extraction

Authors (GJK and AJ) conducted independent data extraction for all studies included. A data extraction template was developed based on the Cochrane Consumers and Communication Data Extraction Template for Included Studies [47]. Data on study characteristics were extracted, including author and date of publication, location and setting of study, reported conflict of interests, study design, statistical methods for analysis, sample size, inclusion and exclusion criteria, psychometric properties of outcome measures, key findings, and limitations. Baseline caregiver characteristics including gender, caregiver age, employment and marital status, relationship to individual and level of education were obtained.

### Evaluation of methodological quality and risk of bias

Risk of bias was assessed using the Quality Assessment Tool for Observational Cohort and Cross-Sectional Studies [48] and recommendations from the Joanna Briggs Institute [49]. Authors (GJK & AJ) independently assessed all included studies in the final review. Disputes regarding the methodological quality of articles were resolved via author discussion.

### Synthesis of results

Narrative synthesis was used to summarise and explain key findings both within and between studies included. Aggregate data and key findings were summarised in tables to include study and sample characteristics, measures used, and key results. The conduct of this review was based on the Preferred Reporting Items for Systematic Reviews and Meta-Analyses guidelines (PRISMA [50];) and the Synthesis without Meta-Analysis in Systematic Reviews guidelines (SWiM [51];).

## Results

### Literature search strategy

**Fig. 1:**
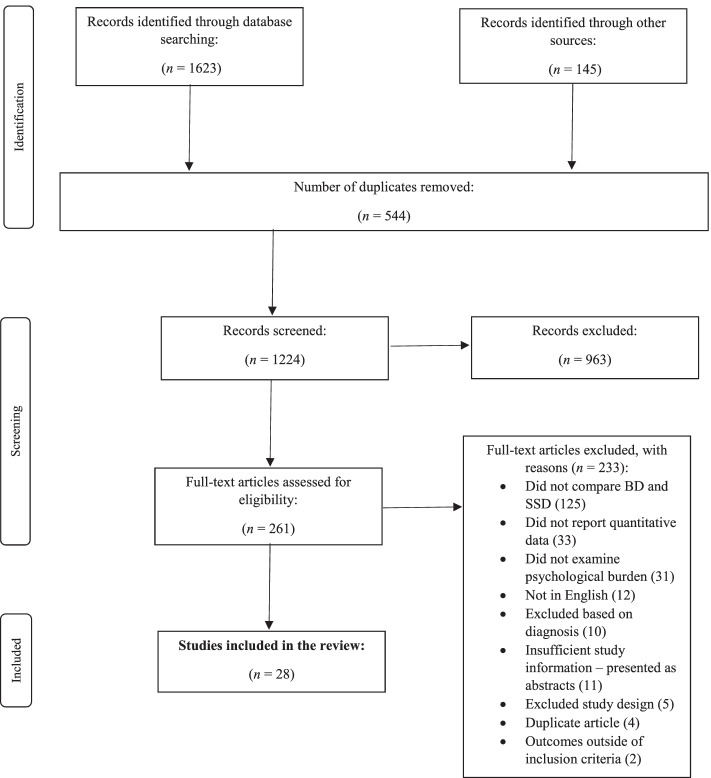
Flow Diagram of The Study Selection Process

The literature search processes are summarised in Fig. 1. Twenty-eight studies met criteria for full-text inclusion.

### Study characteristics

Twenty-five (89.3%) studies were cross-sectional (see Table 1). Recruitment sites included psychiatric hospitals (50%, *n* = 14), university teaching settings (17.8%, *n =* 5), outpatient mental health settings (28.6%, *n* = 8), a transit home (3.6%, *n* = 1), and an unspecified source (3.6%, *n* = 1). Studies were conducted in Asia (57.1%, *n* = 16), Africa (17.9%, *n =* 5), the Americas (10.7%, *n* = 3), Europe (10.7%, *n =* 3), and the Middle East (3.6%, *n* = 1).

**Table 1:** Summary of twenty-eight studies included in this review

Study ID and Authors	Country & Setting	Study Design	Caregiving domains measured	*t*-test, *p* and Cohen’s *d* derived from *M*(*SD*) of SSD and BD comparisons	Key Findings
1. Blanthorn-Hazell et al. [52]	Germany, Spain & UK, community mental health	Cross-sectional	Burden	*t*(240) = 0.30, *p =* .764, *d* = 0.04	- Caring for those with either SCZ or BD with agitation confers substantial and similar burden.
2. Chadda et al. [53]	India, psychiatric outpatient clinic	Prospective cohort	Burden	*t*(198) = 0.78, *p =* .437, *d* = 0.11	- Both caregiver groups experienced similar levels of burden, with no significant differences between caregiving groups.
3. Chakrabarti and Gill [54]	India, psychiatric hospital	Cross-sectional	Burden	*t*(56) = 3.42, *p* < .01, *d* = 0.88	- Total burden scores were significantly greater for caregivers of those with SCZ than BD.
4. Chakrabarti et al. [55]	India	Cross-sectional/ psychometric validation	Distress	Information not available	- There were no differences in distress between caregivers of those with SCZ and BD.
5. Chang et al. [56]	Taiwan, four hospitals	Cross-sectional	Burden, Depression, Anxiety	No total burden score available Depression Total Score *t*(298) = 0.98, *p =* .326, *d* = 0.12 Anxiety Total Score *t*(298) = 0.72, *p =* .470, *d* = 0.09	- There were no significant differences in burden experienced between both caregiver groups. - No significant differences between groups on measures of depression and anxiety.
6. Chien et al. [57]	Hong Kong & China, two psychiatric outpatient clinics	Cross-sectional/ psychometric validation	Burden	*t*(128) = 0.40, *p =* .690, *d* = 0.11	- Level of family burden was not statistically significant between caregiver groups.
7. Fekih-Romdhane et al. [58]	Tunisia, psychiatric hospital	Cross-sectional	Burden	*t*(43) = 1.99, *p =* .053, *d* = 0.61	- No significant differences in burden experienced between caregivers based on diagnosis.
8. Grover et al. [59]	India, tertiary-care teaching hospital	Cross-sectional	Burden, Distress	Total Burden score *t*(138) = 2.92, *p* < .01, *d* = 0.49 Distress comparison data not available	- Overall negative caregiving experience was higher in SCZ group compared to BD. - Positive personal caregiver experience was significantly higher in caregivers of those with SCZ compared to BD. - No significant differences between caregivers on measure of psychological distress.
9. Grover et al. [60]	India, psychiatry outpatient clinic	Cross-sectional	Burden	Information not available	- Caregivers of those with SCZ had significantly higher moderate to severe objective burden compared to caregivers of those with BD. - Caregivers of those with BD has significantly higher subjective burden compared to caregivers of those with SCZ.
10. Grover et al. [61]	India, psychiatry hospital	Cross-sectional	Burden	Objective Burden Score *t*(98) = 1.63, *p =* .107, *d* = 0.33 Subjective Burden Score *t*(98) = 3.67, *p <* .001, *d* = 0.74	- Caregivers in SCZ group had significantly higher objective burden, subjective burden, and disruption to routines.
11. Grover et al. [62]	India, 14 national mental health centres	Cross-sectional	Distress	*t*(1049) = 1.28, *p* = .200, *d* = 0.09	- Caregivers of those with SCZ had significantly higher distress compared to caregivers of individuals with BD.
12. Ak et al. [63]	Turkey, inpatient psychiatric unit	Cross-sectional	Burden	Information not available	- Caregiving burden was high across both groups, but no statistical difference was found.
13. Nehra et al. [64]	India, hospital	Cross-sectional	Burden	Total Objective Burden Score *t*(98) = 0.97, *p* = .334, *d* = 0.19	- All differences were non-significant between the two groups.
14. Ramírez et al. [65]	Colombia, Mood Disorders and Psychosis Clinic	Randomized-controlled trial	Burden	Information not available	- When comparing scores between the BD and schizophrenia groups, no statistically significant differences were found.
15. Rodrigo et al. [66]	Sri Lanka, psychiatric hospital unit	Prospective cohort and cross-sectional	Burden, Depression	Information not available	- No significant differences between groups based on individual diagnosis were found on measures of burden and depression.
16. Roychaudhuri et al. [67]	Nigeria, psychiatric teaching hospital	Cross-sectional	Burden	Total Objective Burden Score *t*(52) = 0.73, *p* = .467, *d* = 0.20 Subjective Positive Appraisal *t*(52) = 1.65, *p* = .104, *d* = 0.45 Subjective Negative Appraisal *t*(52) = 2.23, *p* < .05, *d* = 0.62	- Statistically significant differences between caregivers of those with SSD and BD were only observed in subjective negative burden domain.
17. Sharma et al. [68]	Nepal, psychiatric inpatient unit	Cross-sectional	Burden, Depression, Anxiety	Burden Total Score *t*(98) = 0.48, *p* = .632, *d* = 0.09 Depression Total Score *t*(98) = 0.57, *p* = .572, *d* = 0.11 Anxiety Total Score *t*(52) = 0.67, *p* = .504, *d* = 0.13	- There were no statistically significant differences on burden, depression and anxiety based on individual diagnosis alone. - Spouses of individuals with SCZ had significantly higher stress levels than spouses of individuals with BD.
18. Singh and Prajapati [69]	Nepal, transit home	Cross-sectional	Burden	Information not available	- Moderate caregiving burden was statistically higher in caregivers of those with SCZ compared to caregivers of those with BD.
19. Vasudeva et al. [70]	India, tertiary care hospital	Cross-sectional	Burden	*t*(101) = 2.15, *p* < .05, *d* = 0.42	- Both caregiver groups experience considerable burden, but global burden in caregivers of those with SCZ was significantly higher than in BD group.
20. Webb et al. [71]	USA, hospital inpatient and outpatient programs	Cross-sectional	Burden, Psychological wellbeing	Total Burden Score *t*(82) = 1.45, *p* = .151, *d* = 0.33 Total Wellbeing Score *t*(82) = 1.97, *p* = .052, *d* = 0.17	- No significant differences related to diagnosis of individual were found.
21. Zendjidjian et al. [72]	France, psychiatric hospital unit	Cross-sectional	Mental Health	Mental Health Composite Score *t*(359) = 2.23, *p* < .05, *d* = 0.24	- Caregivers of individuals with SCZ reported significantly lower mental health outcome scores compared to caregivers of those with BD.
22. Zhou et al. [73]	China, nine psychiatric units	Cross-sectional	Burden, Depression, Anxiety	Information not available	- Caregivers of those with BD reported greater burden, regarding violent and suicidal behaviours from individuals, compared to caregivers of those with SCZ.
23. Abdeta and Desalegn [74]	Ethiopia, university hospital	Cross-sectional	Presence of common mental disorders	Information not available	- No significant differences were noted in the percentage of caregivers presenting with common mental disorders (anxiety and depression).
24. Cohen et al. [75]	Brazil, outpatient teaching hospital	Cross-sectional	Mental health, Depression, Psychological outcomes	Mental Health Composite score *t*(123) *=* 0.97, *p* = 0.332, *d =* 0.17 Psychological Outcomes score *t*(123) *=* 1.02, *p* = 0.311, *d =* 0.18 Total Depression score *t*(123) *=* 2.92, *p* < 0.01, *d =* 0.52	- Caregivers of individuals with SCZ had significantly higher depressive symptoms compared to caregivers of those BD. - No significant differences were observed on the mental health and psychological outcome scores between caregiver groups.
25. Ukpong and Ibigbami [76]	Nigeria, outpatient psychiatric clinics	Cross-sectional	Burden, Depression, Anxiety, Psychological outcomes	Total Burden score *t*(198) *=* 5.19, *p* > 0.001, *d =* 0.73 Total Anxiety score *t*(198) *=* − 0.17, *p* = 0.867, *d =* 0.02 Total Depression score *t*(198) *=* 3.25*, p* < 0.001*, d =* 0.46 Total Psychological outcomes score *t*(198) *= 2.62*, *p* < 0.01, *d =* 0.37	- Burden was significantly higher in caregivers of individuals with SCZ compared to caregivers of those with BD. - Caregivers did not significantly differ in anxiety experienced. - Caregivers of those with BD reported significantly higher depression scores than caregivers of those with SCZ. - Caregivers of those with SCZ reported significantly lower psychological outcome scores compared to caregivers of those with BD.
26. Udoh et al. [77]	Nigeria, neuropsychiatric hospital	Cross-sectional	Burden, Psychological Distress	Burden Total Score *t*(128) = 2.39, *p <* 0.05, *d* = 0.45 Psychological Distress Total Score *t*(128) = 0.48, *p* = 0.63, *d* = 0.08	- No significant differences in caregiver burden were reported between caregivers of individuals with SCZ or BD. - Caregivers of individuals with SCZ reported higher levels of psychological distress compared to caregivers of those with BD.
27. Asl et al. [78]	Iran, university psychiatric institute	Descriptive analytical	Burden, Depression, Anxiety, Psychological distress	Total Burden Score *t*(298) = 0.45, *p* = 0.65, *d* = 0.05 Depression, Anxiety and Stress Composite Score *t*(298) = 0.02, *p* = 0.99, *d* < 0.01	- No significant differences in caregiver burden were observed between caregivers of individuals with SCZ or BD. - No significant differences in depression, anxiety or distress were observed between caregivers of individuals with SCZ or BD.
28. Khatoon et al. [79]	India, psychiatric hospital	Cross-sectional	Burden	Total Objective Burden Score *t*(58) = 2.06, *p <* 0.05, *d* = 0.53	- Caregivers of those with SCZ presented with significantly higher caregiver burden on subscales of total objective burden, subjective burden, financial burden, disruption to family activities, disruption to family leisure and disruption to family interaction. - There were no significant differences in caregiver burden observed between caregivers of individuals with SCZ or BD on the clinician-rated objective burden subscale, and burden subscales on effects on physical and mental health.

*SSD* schizophrenia spectrum disorder, *SCZ* schizophrenia, *BD* bipolar disorder, *M* mean, *SD* standard deviation

### Sample characteristics

Across 27 studies, data were presented for 6166 caregivers (see Table 2), with one study omitting caregiver sample size [65]. Sample sizes in studies ranged from 40 caregivers to 1403 caregivers, and nine studies (32.1%) included samples of under 100 caregivers. Across 6166 caregivers, 32.7% (*n* = 2018) were caregivers of individuals with bipolar disorder, 47.9% (*n =* 2955) were caregivers of individuals with schizophrenia spectrum disorders and 19.4% (*n =* 1193) were caregivers of individuals with other mental health conditions. Based on data from 21 studies, 48.6% (*n =* 2997) of caregivers were female. Reporting of caregiver relationships to cared-for individuals varied, but available data indicated 32.3% (*n =* 1993) of caregivers were parents, 24.4% (*n =* 1507) were partners, 12.4% (*n =* 766) were siblings, 4.3% (*n =* 265) were children, and 11.9% (*n =* 732) had other relationships. Data on characteristics related to employment varied; eight studies did not report employment characteristics. Across 20 studies, 56.5% (*n =* 2662) of caregivers held either part- or full-time employment, and 10.7% (*n* = 503) were unemployed. Twenty studies indicated that 58.8% (*n =* 3623) of caregivers were married or in a relationship, 8.2% (*n =* 503) of caregivers were single, 2.3% (*n =* 139) of caregivers were divorced or widowed, and 0.2% (*n =* 13) were listed as “Other”. Employment data on the remaining 55.0% of caregivers, and marital status data on the remaining 30.5% of caregivers, in the overall review sample were not presented.

**Table 2:** Sample characteristics across twenty-eight studies included in this review

Study ID and Authors	*n* (%) of Caregivers (SSD, BD & Other Groups)			Age *M(SD)*	*n* (%) female
	SSD *n*(%)	*BD n(%)*	Other *n*(%)		
1. Blanthorn-Hazell et al. [52]	138(46.0%)	159(54.0%)	N/A	Total *N:* 44.8(13.1)	Total *N* = 214(72.0%)
2. Chadda et al. [53]	100(50.0%)	100(50.0%)	N/A	75.0% between 25 and 50 years of age	SSD = 51(51.0%) BD = 42(42.0%)
3. Chakrabarti and Gill [54]	20(34.5%)	38(65.5%)	N/A	SSD = 48.7(10.7) BD = 39.2(9.3)	SSD = 12(60.0%) BD = 17(45.0%) Total *N* = 29(50.0%)
4. Chakrabarti et al. [55]	20(50.0%)	20(50.0%)	N/A	SSD = 44.7(13.1) BD = 41.7(12.9) Total *N* = 43.2(13.1)	SSD = 8(40.0%) BD = 9(45.0%) Total *N* = 17(42.5%)
5. Chang et al. [56]	215(46.8%)	85(18.5%)	MDD: 159 (34.6%)	SSD = 55.0(13.5) BD = 51.4(12.6) MDD = 52.1(13.8)	SSD = 118(55.0%) BD = 43(51.0%) MDD = 74(47.0%) Total *N* = 235(51.2%)
6. Chien et al. [57]	168(64.1%)	12(4.6%)	Dep: 48 (18.3%)	Total *N* = 42.6(10.8)	Total *N* = 158(60.3%)
7. Fekih-Romdhane et al. [58]	20(38.5%)	25(48.1%)	SCZAF: 7 (13.5%)	Total *N* = 48.4(13.1)	Total *N* = 41(78.8%)
8. Grover et al. [59]	70(50.0%)	70(50.0%)	N/A	SSD = 49.9(11.8) BD = 42.6(13.8)	SSD = 21(33.3%) BD = 40(57.2%) Total *N* = 61(43.6%)
9. Grover et al. [60]	65(53.3%)	57(46.7%)	N/A	Total *N* = 47.1(13.7)	Total *N* = 47(38.5%)
10. Grover et al. [61]	50(50.0%)	50(50.0%)	N/A	SSD = 50.3(14.9) BD = 45.0(13.2)	SSD = 15(30.0%) BD = 26(52.0%) Total *N* = 41(41.0%)
11. Grover et al. [62]	707(50.4%)	344(24.5%)	Dep: 352 (25.1%)	Total *N* = 44.6(12.5)	Total *N* = 620(44.2%)
12. Ak et al. [63]	40(50.0%)	40(50.0%)	N/A	Not reported	SSD = 30(75.0%) BD = 25(62.5%) Total *N* = 55(68.8%)
13. Nehra et al. [64]	50(50.0%)	50(50.0%)	N/A	SSD = 43.50(9.8) BD = 40.1(11.4)	SSD = 23(46.0%) BD = 23(46.0%) Total *N* = 44(44.0%)
14. Ramírez et al. [65]	Not specified	Not specified	N/A	Not reported	Not reported
15. Rodrigo et al. [66]	65(81.3%)	15(18.7%)	N/A	Total *N* = 57.7(13.3)	Total *N* = 44(55.0%)
16. Roychaudhuri et al. [67]	30(55.5%)	24(44.4%)	N/A	Reported as *n*(%): < 35 years = 24 (44.4%) > 35 years = 30 (55.6%)	Total *N* = 20(37.0%)
17. Sharma et al. [68]	50(50.0%)	50(50.0%)	N/A	Total *N* = 43(12.0)	SSD = 23(46.0%) BD = 18(36.0%) Total *N* = 41(41.0%)
18. Singh and Prajapati [69]	40(50.0%)	40(50.0%)	N/A	SSD Carer females = 58.3(9.5) BD Carer females = 0.0(16.7)	SSD = 30(75.0%) BD = 20(50.0%) Total *N* = 50(62.5%)
19. Vasudeva et al. [70]	52(50.5%)	51(49.5%)	N/A	SSD = 48.3(11.7) BD = 47.4(12.0)	SSD = 20(38.5%) BD = 23(45.1%) Total *N* = 43(41.8%)
20. Webb et al. [71]	59(70.2%)	25(29.8%)	N/A	Total *N* = 56.0(13.6)	Total *N* = 66(78.0%)
21. Zendjidjian et al. [72]	246(51.5%)	115(24%)	Dep: 117(24.5%)	BD & MDD Total = 52.2(15.5)	Total *N* = 135(58.2%)
22. Zhou et al. [73]	243(54.9%)	200(45.2%)	N/A	SSD = 47.7(13.7) BD = 44.4(13.6)	SSD = 107(44.0%) BD = 102(51.0%) Total *N* = 209(47.2%)
23. Abdeta and Desalegn [74]	80(37.2%)	60(27.9%)	Dep: 50(23.3%) Anx: 25(11.6%)	Total *N* = 35(1.62)	Total *N* = 120(55.8%)
24. Cohen et al. [75]	63(50.4%)	62(49.6%)	N/A	SSD = 51.28(12.65) BD = 40.55(15.17)	SSD = 52(84.1%) BD = 43(69.4%)
25. Ukpong and Ibigbami [76]	100(50%)	100(50%)	N/A	SSD = 56.13(12.99) BD = 43.03(13.06)	SSD = 63(63%) BD = 52(52%)
26. Udoh et al. [77]	84(20.2%)	46(11.1%)	Dep: 104(25.1%) SUD: 92(22.2%) Others: 89(21.4%)	15–29 years = 181 (43.6%) 30–44 years = 154 (37.1%) 45–59 years = 63(15.2%) Above 60 years = 17(4.1%)	Total female = 236(56.9%)
27. Asl et al. [78]	150(33.33%)	150(33.33%)	ASD: 150(33.33%)	SSD = 42.22(1.28) BD = 48.41(1.37)	SSD = 81(54%) BD = 87(58%)
28. Khatoon et al. [79]	30(50.0%)	30(50.0%)	N/A	20–30 years = 16(26.6%) 30–40 years = 34(56.6%) 40–50 years = 10(16.6%)	Not reported

*SSD* Schizophrenia Spectrum Disorders, *BD* Bipolar Disorder, *SCZAF* Schizoaffective Disorder, *Dep* Depression, *Anx* Anxiety, *SUD* Substance Use Disorders, *ASD* Autism Spectrum Disorder, *M* Mean, *SD* Standard Deviation, *N/n* Number of participants

### Study quality

All studies implemented established measures of caregiver burden and psychological impacts but presented inconsistent detail in their psychometric properties. Caregivers were recruited mostly from psychiatric hospital departments and mental health clinical settings, decreasing the likelihood of sample representativeness for caregivers in the general community. Six studies [52, 66, 69, 74, 77, 78] did not stipulate what diagnostic framework was used to determine diagnosis of individuals. Only four studies [63, 65, 70, 76] used diagnostic assessments to confirm diagnoses of cared-for individuals. Cultural diversity in samples of caregivers was captured given the broad diversity of countries included.

In appraising studies, eleven were of “Higher” quality [52, 54, 56–58, 72, 73, 75, 76], 16 were of “Moderate” quality [53, 55, 59–65, 67–71, 78, 79], and three were of “Lower” quality due to unclear inclusion criteria [66, 74, 77].

### Measurement of caregiver burden and psychological functioning

Measures used to assess caregiver burden and psychological functioning are presented in Table 3. Caregiver burden was assessed using 11 different measures across 23 studies. The Family Burden Interview Schedule [FBIS; 80] was most commonly used across eight studies [57, 60, 61, 64, 67, 69, 76, 79]. There were six measures of objective and/or subjective burden in 18 studies (61.5%), two measures of stress-appraisal coping in four studies (15.4%), two measures of general family burden in two studies (7.7%) and one measure of caregiver strain in two studies (7.7%).

**Table 3:** Measures used across twenty-eight studies to assess caregiver burden and psychological functioning

Name of measure	How construct is defined	How construct is assessed by measure	Studies used in
Burden			
Involvement Evaluation Questionnaire (IEQ [80];)	Stress-appraisal-coping model	29-items across four factors: tension, supervision, worrying and urging. A total score represents overall burden.	1, 9, 10
Burden Assessment Schedule (BAS [81];)	Objective and subjective burden	40-items across nine factors: spouse (caregiver related), physical & mental health of caregivers, external support, caregiver’s routine, support of individual, taking responsibility, other relations, individual’s behaviour, and caregiver strategy. A total score represents overall burden.	2, 3, 19
Caregiving Burden Inventory (CBI [82];)	Burden defined through the five factors assessed	24-items across five factors: time-dependent; developmental; physical; social and emotional.	5
Family Burden Interview Schedule (FBIS [83];)	Objective burden with a subjective burden question included.	24-items across six factors: financial burden, disruption of family routine, disruption of family leisure, disruption of family interactions, effect on physical health and mental health of relatives. One additional item assessing subjective burden. Two total scores representing objective and subjective burden. Clinician-rated.	6, 9, 10, 13, 16, 18, 25, 28
Subjective Well-being Inventory (SUBI [84];)	Subjective burden	Two sets of items assessing positive and negative assessments of well-being and subjective burden. Two total scores for positive and negative sets.	16
Zarit Burden Interview (ZBI [85];)	Subjective burden	12-items assessing caregiver’s state of health, psychological and financial wellbeing, social life, and relationship with the individual. A total score represents overall subjective burden.	7, 12, 26, 27
Modified Caregiver Strain Index (MCSI [86];)	Caregiver strain	13-items across six factors: social, psychological, physical, time, financial and employment. Higher scores indicate greater caregiver strain.	15, 17
Experience of Caregiving Inventory (ECI [87];)	Stress-appraisal-coping model	66-items across ten factors: eight negative aspects of caregiving (difficult behaviours, negative symptoms, stigma, problems with services, effects on family, need for back-up, dependency, and loss) and two positive aspects of caregiving (positive personal experience and good aspects of relationship). Three total scores represent overall negative score, overall positive score, and global score.	8
Family Burden Schedule (adaptation of the Spanish adaptation of the Social Behaviour Assessment Schedule) (FBS [88];)	Objective burden, attribution level and subjective burden.	Number of items not specified. Assesses burden across three factors: objective burden evaluates change in daily functioning; level of attribution evaluates whether caregivers consider their problems related to the individual; subjective burden evaluates perception of stress related to subject’s behaviour.	14
Significant Other Scale (based on subscale of Family Evaluation Form) [89]	Objective burden	37-item measure, with the study using a 13-item subscale described by the original measure authors as assessing objective burden. One total score representing overall burden.	20
Name of measure	How construct is defined	How construct is assessed by measure	Studies used in
Family Experience Inventory Schedule (FEIS [90];)	Family burden + Depression and anxiety	28-items across five factors: violent behaviour, depression and anxiety symptoms and social isolation of the caregiver, disruption of caregiver routines, individual suicidality, and satisfaction with the quality-of-service provision.	22
Psychological Functioning			
Distressing Symptom Rating Scale (DSRS [55];)	Caregiver distress	44-items across 16 factors: depressive/negative, manic, socially disruptive, somatic, delusions/odd beliefs, hallucinations, cognitive, aggression, work, self-care, dependence, compliance, side effects, substance abuse, insight, poor rapport, and other.	4
Taiwanese Depression Questionnaire (TDQ [91];)	Presence and severity of depression	18-items evaluating depressive symptoms over past week, calculated to obtain total depression score.	5
Beck Anxiety Inventory (BAI [92];)	Presence and severity of anxiety	21-items summed to obtain total anxiety score.	5, 17
General Health Questionnaire (GHQ [93];)	Psychological morbidity/distress	12-items summed up obtain total score assessing psychological distress/morbidity.	8, 11, 26
Center for Epidemiological Studies – Depression Scale (CES-D [94];)	Possibility of depression	20-items with one total score representing overall depression.	15
Beck Depression Inventory (BDI [95];)	Presence and severity of depression	21-items with one total score representing depression.	17, 24
National Center for Health Statistics General Wellbeing-Schedule (from the Medical History Questionnaire) [96]	Wellbeing	22-items across six factors of caregiver wellbeing: anxiety, depression, general health, positive wellbeing, vitality, self-control.	20
Family Experience Inventory Schedule (FEIS [90];)	Family burden + Depression and anxiety	28-items across five factors: violent behaviour, depression/anxiety symptoms and social isolation of the caregiver, disruption of caregiver routines, individual suicidality, and satisfaction with the quality-of-service provision.	22
Self-Reported Questionnaire-20 (SRQ-20 [97];)	Presence of common mental disorders (anxiety and depression)	20-item screening tool with one total score designed to assess the presence of symptoms of common mental disorders, such as anxiety and depression.	23
Short Form-36 (SF-36 [98];)	Quality of life (including mental health outcomes)	36-items across eight factors: physical functioning, social functioning, role-physical problems, role-emotional problems, mental health, vitality, bodily pain, and general health. Two component summary scores representing physical and mental overall score.	21, 24
World Health Organization Quality of Life Instrument – BREF (WHOQOL-BREF [99];)	Quality of life (including psychological outcomes domain)	26-items divided into four domains: physical, psychological, social relationships and environment, along with two general questions of quality of life. Total scores range from 0 to 100 and the higher the value of scores, the higher quality of life.	24, 25
Hospital Anxiety and Depression Inventory (HADS [100];)	Anxiety and depression	14-item measure of anxiety and depressive symptom severity. One total score is calculated with higher scores representing greater symptom severity.	25
Depression, Anxiety and Stress Scale (DASS [101];)	Depression, anxiety, and stress	42-item measure of three 14-item subscales of depression, anxiety, and stress. Three total scores representing each domain are calculated measured from mild to severe severity.	27

Psychological functioning was assessed using 13 measures across 14 studies. Nine measures were used to assessed anxiety, depression or both [90–92, 94, 95, 97, 100, 101], two assessed variations of caregiver distress [55, 93] and two measured quality of life (QoL) including a specific mental health or psychological outcomes domain [98, 99]. Psychological wellbeing [96] was directly assessed in one study [71], while one study [59] implemented the Experience of Caregiving Inventory (ECI [87];), a stress-appraisal-coping measure of caregiving burden that examines both the positive and negative experiences of providing care.

### Comparison of caregiver burden

#### No significant differences

Fourteen (50%) studies comparing caregiver burden, comprising 2091 caregivers [52, 53, 56–58, 63–66, 68, 71, 77–79], showed no significant differences in caregiver burden between caregivers of individuals with bipolar disorder or schizophrenia spectrum disorders. Two studies [77, 78] presented data on caregivers of those with schizophrenia and bipolar disorder as part of a larger group; relevant data was extracted and *t*-tests were re-ran to determine differences in caregiving burden observed. Studies emphasized that despite the perceived episodic nature of bipolar disorders, greater chronicity and severity of symptoms across both diagnoses may confer similar burden [52, 53, 58, 63, 68, 78]. Caregiver characteristics were associated with burden across both groups, including lower level of education attained, being a spousal caregiver, and higher caregiver distress.

#### Significantly greater caregiver burden in schizophrenia spectrum disorders

Nine (32.1%) papers comprising 1217 caregivers demonstrated that caregiving burden was significantly greater in caregivers of individuals with schizophrenia spectrum disorders than in caregivers of individuals with bipolar disorders [59–61, 67, 69, 70, 76, 79]. Five studies noted significantly lower functioning in participants with schizophrenia as compared to participants with bipolar disorder, but this was inconsistently associated with heightened caregiver burden [54, 59–61, 70]. Other studies noted that individuals diagnosed with schizophrenia presented with more severe and longer duration of untreated illness compared to individuals diagnosed with bipolar disorders [54, 70, 79]. This was not uniform across studies, as Grover et al. [59] reported longer duration of treated illness for participants with bipolar disorder compared to individuals with schizophrenia (Bipolar Disorder, *M* = 160.06, *SD* = 97.50; Schizophrenia, *M* = 112.82, *SD* = 74.07, *t* = 3.24, *p* = .001). Individuals with bipolar disorder may experience more defined periods of well and ill-health compared to the persistent functional and symptomatic impacts of schizophrenia. However, this would not hold true for individuals with chronic and persistent bipolar disorder [54].

Several caregiver and illness characteristics were proposed as contributing to the greater burden experienced by caregivers of people with schizophrenia. Chakrabarti and Gill [54] noted that caregiver burden (Schizophrenia Spectrum Disorders, *M* = 71.95, *SD* = 14.09; Bipolar Disorders *M* = 61.42, *SD* = 9.26; *t* = 3.42, *p* < .01) was greater for caregivers of those with schizophrenia in all burden domains except for that pertaining to the relationships caregivers have with other family members or friends. While no discernable reason was reported for this lack of difference regarding other relationships, high burden was correlated with several coping strategies. Caregivers of individuals with schizophrenia were more likely to adopt avoidance, resignation and seeking spiritual help coping as opposed to caregivers of individuals with bipolar disorder. Grover et al. [61] noted that caregivers of those with schizophrenia believed their diagnosed loved ones had higher unmet healthcare needs than caregivers of those with bipolar disorder. These unmet needs included amelioration of psychotic symptoms, need for psychoeducation and relief of psychological distress. Caregivers with heightened burden also presented with higher levels of psychological distress. Vasudeva et al. [70] found that caregivers of those with schizophrenia had significantly higher total objective burden score (*t* = 2.15, *p* < .05, *d* = 0.42), higher burden in needs for external support (*p* < .05, *d* = 0.48), disruptions to caregivers’ routine (*p* < 0.01, *d* = 0.539), and higher scores in other relations (*p* < .05, *d* = 0.38). Whilst these differences were attributed to the ongoing functional and symptomatic impacts of schizophrenia, the study authors posited that caregivers of those with schizophrenia may have ongoing concerns about their loved one’s capacity to return to normal functioning, even when acute symptoms were treated.

#### Significantly greater caregiver burden in bipolar disorders

Two (7.7%) studies comprising 565 caregivers demonstrated that caregiving burden was significantly higher in caregivers of individuals with bipolar disorder compared to individuals with schizophrenia [60, 73]. Zhou et al. [73] demonstrated that caregiver perceptions of violent behaviour (*B* = 2.01, *p* < .001) and suicidal risk (*B* = 0.51, *p* < .05) were greater in caregivers of individuals with acute bipolar disorder compared to caregivers of those with acute schizophrenia spectrum disorders. The focus on acute presentations of illness may explain the outcomes obtained, but symptom severity comparison was not possible due to differences in symptom measures used. Grover et al. [60] noted that caregivers of those with bipolar disorder appraised their caregiving burden to be higher than caregivers of those with schizophrenia on the Involvement Evaluation Questionnaire (IEQ; *t* = 2.96, *p* < .01). While direct explanations were not provided, considerations of how caregiver characteristics influence caregiver burden was emphasized given its multidimensionality.

### Comparison of psychological functioning

#### No significant difference in mental health outcomes

Eleven (39.3%) studies comprising 3246 caregivers demonstrated no significant differences in mental health outcomes for caregivers associated with caring for individuals with either schizophrenia spectrum disorders or bipolar disorders [55, 56, 59, 62, 66, 68, 73–76, 78]. One study [78] presented data on caregivers of those with schizophrenia and bipolar disorder as part of a larger group; relevant data was extracted and *t*-tests were re-ran to determine differences in mental health outcomes observed. The studies demonstrated that while caregivers experienced high levels of distress, depressive and anxious symptoms, diagnosis did not differentiate outcomes. Many studies noted the likelihood that both diagnoses confer similar distress to caregivers given their chronicity, symptom severity and functional impacts. Certain caregiver characteristics demonstrated mixed associations with poorer mental health outcomes, including caregiver substance use, lower caregiver educational status, heightened caregiver burden, caregivers experiencing illness themselves, and being a spousal caregiver.

#### Significant mental health outcomes in caregivers of those with schizophrenia spectrum disorders

Four studies (11%) comprising 933 caregivers demonstrated poorer mental health outcomes of caregivers of those with schizophrenia spectrum disorders compared to caregivers of those with bipolar disorder [72, 75–77]. One study [77] presented data on caregivers of those with schizophrenia and bipolar disorder as part of a larger group; relevant data was extracted and *t*-tests were re-ran to determine differences in mental health outcomes observed. Zendjidjian et al. [72] found that caregivers of those with schizophrenia reported significantly lower scores on the mental health domains of a quality-of-life measure, the Short Form-36 (SF-36 [98];). Whilst no direct reason was offered to explain these differences, this may be indicative of caregiving as multidimensional, and suggests that caregiver supports should be tailored to address the most-impaired domains within a caregiver’s experience. Cohen et al. [75] noted that caregivers of those with schizophrenia presented with higher depressive symptoms, attributing this to positive psychotic symptoms and stigma, which are more commonly associated with schizophrenia. Ukpong and Ibigbami [76] demonstrated that caregivers of those with schizophrenia had poorer mental health. Factors associated with poorer mental health across both caregiving groups included older caregiver age, longer duration of illness and caregiving, caregivers not being married, and increased caregiver burden, anxiety, and depression.

#### Significant mental health outcomes in caregivers of those with bipolar disorders

One study (3.8%) comprising 200 caregivers demonstrated poorer mental health outcomes in caregivers of those with bipolar disorder when compared to caregivers of individuals with schizophrenia [76]. The study demonstrated that caregivers of individuals with bipolar disorder presented with higher depressive symptoms than caregivers of those with schizophrenia. This finding was unexpected, and significant negative correlations were noted between caregiver depressive symptoms and all four domains of the QoL measure used, which assessed physical, psychological, social, and environmental QoL.

#### Comparison of psychological wellbeing

Only two studies, comprising 224 caregivers, examined outcomes pertaining to psychological wellbeing of caregiving [59, 71]. Webb et al. [71] assessed caregiver wellbeing but found no significant differences based on individual diagnosis. This may have been attributable to small sample size and reliance on chart diagnosis. However, older caregiver age and higher frequency of positive symptoms were significantly associated with greater well-being. While reasons for the latter were not explored, as caregivers age, they may be more accepting of their loved one’s illness or are better equipped to access social supports. Grover et al. [59] noted that caregivers of those with schizophrenia spectrum disorders reported higher positive personal caregiver experiences than caregivers of those with bipolar disorder (*t*(138) = 2.67, *p* < .001) on a measure of stress-appraisal coping. Positive appraisals were significantly correlated with negative care appraisals across both groups. Individuals diagnosed with bipolar disorder had longer illness and treatment duration, which may concurrently reduce their caregiver’s experience of role demands and positive aspects of caring.

## Discussion

To our knowledge, this was the first systematic review that examined whether experiences of caregiver burden and psychological functioning differ for caregivers depending on whether they provide care to an individual diagnosed with a schizophrenia spectrum disorder or bipolar disorder. Previous systematic reviews have focused on examining the negative impacts of caregiving for these two groups separately [41–43] or have examined the broader categorization of SMI without specific diagnostic focus [44, 45].

### The comparative outcomes of the caregiving experience

#### Caregiver burden

A primary finding amongst all studies, except for Singh and Prajapati [69], was that both groups experienced high levels of burden. This is unsurprising, yet consistent with literature noting high caregiver burden experienced by caregivers of those with SMI [37, 102]. Singh and Prajapati [69] attributed this exception to their sample of individuals diagnosed with bipolar disorder not being acutely unwell, but this contrasts the literature indicating that non-acute bipolar disorder still confers symptomatic and functional impacts to diagnosed individuals [103].

Within the 14 studies demonstrating no significant difference in caregiving burden experienced between both caregiver groups, the comparable symptomatic and functional impairments associated with both schizophrenia spectrum disorders, and bipolar disorders, were highlighted. However, individual symptom severity was only directly compared on the same measure in three studies [53, 58, 63], and while higher symptom severity significantly contributed to burden, burden did not differ between caregiver groups based on symptom severity. There were conflicting results on the influence of transdiagnostic symptoms, such as aggression and suicidality, contributing to comparable levels of caregiver burden [52] and to greater caregiver burden in bipolar disorder alone [73]. However, the latter focused on individuals with acute symptom presentations at recruitment. This may indicate that the combination of symptom severity and acuity were of higher relevance to caregiver burden then the diagnosis itself, given this is an isolated finding within the review. Across many studies, groups presented with similar degrees of chronicity [53, 58, 63, 64, 68]. Given both bipolar disorder and schizophrenia spectrum disorders are associated with disabling impacts [104], chronicity of illnesses could contribute to comparable caregiver burden. Whilst transdiagnostic features were not a primary focus, similarities in caregiver burden across groups may warrant an exploration of these features in the caregiver literature. This suggestion is raised given the growing perspective in psychiatric research that traditional psychiatric diagnoses may limit novel developments in clinical and research settings [105, 106]. Additionally, caregiver characteristics were noted as contributing to heightened burden across both groups, emphasizing that caregiving was not only influenced by the individual being cared for and their clinical characteristics.

The nine studies demonstrating higher caregiver burden in caregivers of those with schizophrenia compared to bipolar disorder demonstrate some consistencies. While group characteristics such as illness duration were mostly comparable, two studies noted that individuals with schizophrenia had higher duration of untreated illness [59, 70]. Even when individuals with bipolar disorder had longer illness duration, the significantly higher level of burden in caregivers of those with schizophrenia was attributed to the symptomatic and functional impacts schizophrenia can incur upon affected individuals [79]. The role of functional impairments in contributing to caregiver burden was possible given five studies noted lower functioning in individuals with schizophrenia; however, functioning was not a consistent correlation of, or contributor to, differential caregiver burden. Other potential caregiver characteristics differentiating caregiver burden were suggested, including the greater stigma around schizophrenia [56], appraisal of illness and functional expectations [59, 70], specific caregiving coping [54], unmet caregiver and individual needs [61] and cultural expectations of functioning [67]. Higher levels of caregiving burden for caregivers of those with bipolar disorder were considered with respect to acuity of symptoms [73] and limited clinician knowledge regarding caregiver burden in bipolar disorder [60]. However, few studies supported differentiation in caregiver burden based on diagnosis, contrasting previous literature emphasizing the association between burden, clinical, individual and caregiver characteristics [55, 107, 108].

The current review findings can be considered in a broader context of the literature examining caregivers of those with other medical conditions. Caregiving burden across numerous medical conditions, including cancer [109], dementia [12], neurological conditions [110, 111] and stroke [112], has been demonstrated as consistently high, and is often compared between these conditions. However, the influence of diagnosis alone on caregiving burden in these medical conditions is, similar to this review’s findings, mixed [11, 13, 109]. Emphasis has instead been placed upon the multidimensionality of caregiving burden as influenced by numerous factors. These have included symptom severity and behavioural disturbances across multiple forms of dementia [12, 113], presence of psychiatric symptoms of anxiety and depression across patients with stroke or neurological conditions [110–112], and caregiver factors including the amount of time and effort spent caregiving across caregivers of those with dementia, stroke or cancer [12, 109, 112]. This demonstrates that, for these more commonly researched medical conditions, caregiver burden is influenced by multiple factors that may be common amongst several diagnoses or may not be associated with diagnosis at all.

Considering the current results and the broader literature of caregiving burden in other conditions, these findings suggest that diagnosis alone may not determine caregiver burden experienced by caregivers, similar to a prior systematic review [44]. Instead, caregiving burden is a multidimensional concept, consisting of multiple domains influenced by numerous individual and caregiver factors. These findings suggest that when considering the various factors in the caregiving experience, diagnosis may not be as relevant as other transdiagnostic illness characteristics, or characteristics of the caregiver themselves. Whilst the focus on diagnosis is important given it reflects the widespread implementation in clinical and research settings of these defined psychopathologies, it is possible and worth exploring whether caregivers view diagnosis with the same significance. This is further reflected in the many concepts intermittently considered in this review and the papers within it that are distinct from diagnosis, including expressed emotion [114], caregiver physical health [115], coping strategies [32], quality of life [116] and social supports [17], amongst several others.

#### Conceptualization of caregiving burden

The lack of standardization for caregiver burden means that, despite relatively sound psychometric data, the diversity of measures and definitions limits consistency of how caregiver burden is assessed across studies. The wide variation in this relatively small sample of caregiving studies is reflective of the wider difficulties in operationalizing caregiving burden. A systematic review and meta-analysis of tools used to assess caregiving burden [117] noted that this multidimensionality meant any measure applied would be informed by the burden dimensions specifically examined, and considerations of the study design, including study location. Despite the review noting the Zarit Burden Interview as being the most commonly adopted and psychometrically sound measure of caregiver burden [117], it was only used four times [58, 63, 77, 78] in this current review. Comparatively, the Family Burden Interview Schedule (FBIS) was applied across eight studies in this review. This may reflect that many studies in this review were based in India, where the FBIS is well-validated [54, 60, 61]. The diversity in measures limits global generalizability of results and conclusions that can be drawn in comparing caregiver experiences.

#### Psychological functioning and mental health outcomes

Juxtaposing the findings around caregiving burden, consistency was noted across the 14 studies examining the mental health outcomes of caregiving – that being depression, anxiety, and psychological distress. As eleven studies demonstrated no significant differences in mental health outcomes of caring between caregiver groups based on individual diagnosis, this may reflect that these aspects of caregiving are better understood and operationalized in comparison to burden. Despite overlap in the mental health outcomes measured, anxiety, depression and distress are relatively distinct and well-defined concepts compared to burden, and the findings present more robustly. Most studies acknowledged that the mental health outcomes measured were similarly high for both caregiver groups [73, 74, 76, 78]. The lack of differentiation in mental health outcomes may reflect the similarly impactful nature of bipolar disorder and schizophrenia spectrum disorders to individuals and their caregivers [24, 118, 119]. Both individual and caregiver characteristics were considered, as spousal caregivers experienced greater distress compared to parental caregivers [68]. It is important to consider caregiver characteristics, as caregiving is influenced by factors beyond diagnosis, including poorer quality of life, older caregiver age, longer illness duration and stigma [75, 76].

#### Psychological wellbeing

The two studies in the current review that considered psychological wellbeing and the positive outcomes of care is indicative of the wider need to focus on this under-researched but developing area [30, 31, 120]. The two studies in this review were not consistent in their findings on psychological wellbeing, as one [71] found no difference between groups, while the other [59] noted that caregivers of those with schizophrenia reported higher positive, and paradoxically negative, caregiving experiences. This disparity in findings may reflect the difference in measures used to examine the positive outcomes of care between caregiver groups. Grover et al. [59] explored positive appraisals of care in the context of a stress-appraisal coping measure of burden, whilst Webb et al. [71] implemented a direct measure of psychological wellbeing to assess these positive outcomes. Differences were also noted in the factors that associated with these findings, which may suggest that given the differences in how positive outcomes of care were assessed, these findings were not measuring the same concept. Further research is warranted, as studies outside of this review have noted that positive personal characteristics may mediate the impacts of caring, and proactive resilience strategies may improve caregiver experiences [30, 31].

### Methodological limitations of the current literature

A noteworthy strength of the literature within this review is its cross-cultural nature, with 18 countries identified across the review in both developed and developing nations. However, many studies did not consider whether cultural factors influenced differences in family structures and who may adopt a caregiving role, and whether that influenced differences in caregiving. Most studies were conducted in developing nations, where caregiving demands may be higher due to less developed mental health systems, cultural stigma towards mental illness or differences in cultural practices. This may bias the current review findings, and further research is needed to determine how caregivers in developed and developing nations differ in their caregiving experiences. Several important methodological limitations were identified. First, most studies were cross-sectional, limiting causal inferences. Attempts were made within studies to account for confounding variables, however which variables were considered was inconsistent across studies. Second, variation in frameworks and measures used to assess caregiver burden meant that comparison of results was challenging for this complex and multidimensional construct. This limitation raises concerns regarding conceptual overlap in measures, as some, such as the Family Experience Inventory Schedule, examined caregiving burden by including caregiver anxiety and depression. Additionally, while some studies included significantly large sample sizes ranging from 200 to 1403 caregivers [52, 56, 62, 72, 73], 32.1% of studies had sample sizes below 100 caregivers, limiting generalizability and strength of results. The lack of control groups in all studies except for Zendjidjian et al. [72] limits the extent to which the results can compare caregiving for those with SMI to the typical caregiving demands that arise from relationships irrespective of the presence of SMI or caregiving associated with other chronic health conditions. Finally, all studies, except for Zhou et al. [73], did not examine early stage or acute-phase bipolar disorder and schizophrenia spectrum disorders, omitting explorations of how caregivers at the early stages of illness adapt to their newfound caring role.

### Strengths and limitations of the review

We implemented a comprehensive search strategy across major databases, and all potentially eligible studies were assessed for inclusion and methodological quality by two independent raters. However, there were limitations. First, studies were excluded if they were not written in English. Twelve papers were omitted from full-text analysis due to this limitation, and these studies took place in developed European nations such as Germany, Greece, Switzerland, and Turkey, alongside Japan and China. Scans of abstracts and listed authors indicated that these excluded studies were not reporting on the same cohorts as other papers in this review. This limits the review’s cultural representativeness; however, we did not aim to determine how cultural experiences influences caregiving. Second, while the data extraction template was based on established Cochrane standards [47], two quality appraisal tools were used to appraise article quality, which may compromise validity of the approach used. Thirdly, while the study authors have commented on the possible influence of transdiagnostic features compared to diagnoses, the current review’s deliberate focus on diagnosis limited an exploration of this influence. Fourthly, the current review did not examine quality of relationships between caregivers and their loved ones. While this was not a focus of the review and is often overlooked in caregiving literature, it was considered in some of the included studies [59, 66] and the quality of these relationships are important considerations within the wider familial and social context of any caregiver-patient dyad. Finally, the current review omitted qualitative studies, which represent an important and substantial area of the caregiver literature [32, 102].

### Directions for future research

Future research would benefit from using longitudinal and real time assessment study designs, and employing consistently larger sample sizes and control groups, to assess changes in caregiving over time and accurately compare differential caregiving experiences. The diversity of conceptualizations and measures for caregiver burden limits comparability, and future research should move towards standardizing caregiving burden for conceptual clarity. Optimistically, current research is moving towards establishing commonality in defining caregiving burden [121]. This is sorely needed in the field to allow better comparability across studies. Given the focus on caregivers of those with chronic presentations of schizophrenia spectrum disorders and bipolar disorders, further research could compare caregiving experience at the early stages of illness. This may reveal differential outcomes of caregiving as caregivers are adapting to their newfound role [122]. The relative paucity of comparative research examining the positive experiences of care presents an opportunity to understand the resilience and personal strengths caregivers have. Finally, the growing interest in transdiagnostic features in psychiatric research [105, 106] presents an opportunity in the caregiver literature to go beyond examinations of caregiving in discrete diagnostic groups, and to think more broadly about the experience of caregiving as a whole.

### Clinical implications

There are several clinical implications of these findings. Establishing a consistent definition for the various factors of caregiving that can be reliably assessed is important in aiding clinicians to accurately understand the caregiver role, and what supports caregivers may require. As caregiving is multidimensional, consideration of the evolving needs of caregivers would better inform supports, rather than focusing on how diagnoses affect caregivers. While there are inconsistent findings on whether diagnosis distinguishes experiences of care, it does suggest that both disorders confer significant burden and psychological impacts. Early implementation of caregiver supports, including peer and psychological support, respite, financial or relief services specifically focused on supporting caregivers of loved ones entering mental health care for the first time, may prevent longer-term negative impacts. Psychoeducation has been implemented across settings as a primary support for caregivers, and while modest benefits have been noted [123], further research is required to determine whether certain modalities, such as group or individual interventions, are most beneficial in supporting caregiver health and wellbeing. Finally, clinicians would benefit from being able to provide ongoing support for caregivers, as caregivers’ needs will likely change over time.

## Conclusions

In this review we have considered whether the caregiving experience is comparable with respects to caregiving burden and psychological functioning for caregivers of those diagnosed with either schizophrenia spectrum disorder or bipolar disorder. Twenty-eight studies presented varying results regarding the similarities and differences of these two groups. Most papers suggested that individual diagnosis did not differentiate the caregiving experience. However, a lack of definitional and measurement consensus for caregiving burden means that comparison is difficult. Similarly, several methodological issues were noted which impacts the generalizability of results. We suggest that both caregiver groups experience significant burden and mental health outcomes. Future studies should aim to: (a) incorporate longitudinal and real time assessment study designs with larger samples examining various components of the caregiving role; (b) establish a standardized definition of burden; (c) assess the positive outcomes of caring; and (d) focus on diverse caregiving populations, including cross-cultural and early-stage caregivers, to determine how caregiver and individual factors influence the caring experience.

## Data Availability

Data, search strategies and appraisals of included studies are available from the corresponding author upon request.

## Abbreviations

SMI: Serious Mental Illnesses
M: Mean
SD: Standard Deviation
QoL: Quality of Life
BD: Bipolar Disorders
SSD: Schizophrenia Spectrum Disorders
SCZ: Schizophrenia
IEQ: Involvement Evaluation Questionnaire
BAS: Burden Assessment Schedule
CBI: Caregiver Burden Inventory
FBIS: Family Burden Interview Schedule
SUBI: Subjective Wellbeing Inventory
ZBI: Zarit Burden Interview
MCSI: Modified Caregiver Strain Index
ECI: Experience of Caregiving Inventory
FBS: Family Burden Schedule
FEIS: Family Experience Inventory Schedule
DSRS: Distressing Symptom Rating Scale
BAI: Beck Anxiety Inventory
GHQ: General Health Questionnaire
CES-D: Center for Epidemiological Studies – Depression Scale
BDI: Beck Depression Inventory
SRQ-20: Self-Reported Questionnaire-20
SF-36: Short Form 36
HADS: Hospital Anxiety and Depression Inventory
DASS: Depression, Anxiety and Stress Scale
WHOQOL-BREF: World Health Organization Quality of Life Instrument – Brief
TDQ: Taiwanese Depression Questionnaire
